# Erosive/Abrasive Enamel Wear While Using a Combination of Anti-Erosive Toothbrush/-Paste

**DOI:** 10.3290/j.ohpd.a43352

**Published:** 2020-04-01

**Authors:** Philipp Körner, Deborah S Inauen, Thomas Attin, Florian J Wegehaupt

**Affiliations:** a Resident, Clinic of Preventive Dentistry, Periodontology and Cariology, Center of Dental Medicine, University of Zurich, Zurich, Switzerland. Wrote the manuscript.; b Dental Master Student, Clinic of Preventive Dentistry, Periodontology and Cariology, Center of Dental Medicine, University of Zurich, Zurich, Switzerland. Performed the experiments in partial fulfilment of requirements for a Master’s degree, proofread the manuscript.; c Professor and Director, Clinic of Preventive Dentistry, Periodontology and Cariology, Center of Dental Medicine, University of Zurich, Zurich, Switzerland. Research idea, contributed substantially to discussion, proofread the manuscript.; d Head of Division of Preventive Dentistry and Oral Epidemiology, Clinic of Preventive Dentistry, Periodontology and Cariology, Center of Dental Medicine, University of Zurich, Zurich, Switzerland. Research idea, hypothesis, experimental design, contributed substantially to discussion and writing the paper, proofread the manuscript.

**Keywords:** anti-erosive, dental erosion, dental abrasion, toothbrush, toothpaste

## Abstract

**Purpose:**

To investigate dental enamel wear caused by erosion and abrasion while using a combination of anti-erosive toothbrush/-paste.

**Materials and Methods:**

A total of 60 enamel specimens from bovine incisors were randomly assigned into five groups of 12 specimens each (G1–5, n = 12): (G1) control group (no treatment), (G2) standard medium toothbrush Paro M43 and standard toothpaste Elmex Caries Protection, (G3) standard medium toothbrush Paro M43 and anti-erosive toothpaste Elmex Protection Erosion, (G4) anti-erosive toothbrush Elmex Erosion Soft and standard toothpaste Elmex Caries Protection, (G5) anti-erosive toothbrush Elmex Erosion Soft and anti-erosive toothpaste Elmex Protection Erosion. Initially, surface baseline profiles were recorded using profilometry. In a total of 60 cycles, all specimens were exposed to hydrochloric acid (pH = 3) for 1 min, rinsed with tap water to stop the erosive attack and brushed according to the specific protocol of each group (15 brushing strokes per run). Enamel loss was determined by comparing the surface profiles before and after 60 cycles and the results were statistically analysed using analysis of variance (ANOVA).

**Results:**

The significantly highest loss of enamel was observed in the control group G1(1.4 ± 0.20 µm) (p < 0.001). G2 turned out to be the most abrasive toothbrush/-paste combination (1.12 ± 0.15 µm), G3 the least invasive (0.40 ± 0.04 µm) (p < 0.001, respectively).

**Conclusion:**

All combinations of the investigated toothbrushes/-pastes reduce erosive/abrasive enamel wear. However, the highest reduction was observed for the combination of anti-erosive toothpaste and standard toothbrush (G3).

The loss of dental hard tissues caused by erosion and/or abrasion has been addressed in numerous recent studies and gained increasing attention in modern dentistry. In this context, specific dental healthcare products such as anti-erosive toothpastes or toothbrushes aiming to avoid or reduce erosion and/or abrasion have been developed and brought to market.

Dental erosions are defined as chemically induced surface dissolution of dental hard tissue caused by acids without any involvement of bacteria.^21^ In minerally undersaturated milieus, acids dissolve minerals from tooth surfaces leaving a demineralised and softened surface layer behind which easily can be removed by mechanical forces.^35,42^ Additionally, a continuous layer-by-layer dissolution might appear resulting in a permanent loss of dental hard tissues.^25^ Due to various risk factors, people of all ages face acid exposure in many different ways in their everyday life. The respective acids are either of extrinsic or intrinsic origin. While extrinsic acids are provided mainly in acidic beverages, food and medicaments,^9,18,19^ intrinsic acids have their origin in gastric juice containing hydrochloric acid^28^ which comes into contact with dental hard tissues during vomiting (related to bulimia nervosa)^31^ or reflux (related to gastroesophageal reflux disease).^11^

Besides kind and frequency of acid exposure, the severity and progression of erosive defects are influenced by modifying host factors such as flow rate, buffering capacity, pH value and composition of saliva.^19^

Dental abrasions are defined as the non-carious, mechanically induced loss of dental hard tissues caused by interaction with objects other than tooth–tooth contact.^24^ Besides individual factors such as brushing habits, frequency and force, abrasions have been shown to significantly be related to the abrasiveness of toothpastes (kind, size, shape, amount of abrasive particles).^30^ The abrasive potential of toothpastes is commonly specified as relative enamel (REA) or dentine abrasion (RDA).^16,17^ Additionally, in respect to length, stiffness and shape of bristles, the type of toothbrush used as a paste-carrying and cleaning tool is reported to have a modulating influence.^41^

In human teeth, as well as in bovine teeth, erosively softened enamel has been shown to have increased susceptibility to abrasion caused by mechanical/abrasive forces.^6,7,42^ As a result, hypersensitivities, exposed dentine surfaces and, as long-term effects, loss in vertical dimension, occlusion and aesthetic problems might occur.^22^ In order to avoid or reduce erosive/abrasive dental wear, different preventive measures and approaches including specific dental healthcare products such as anti-erosive toothpastes and toothbrushes have been developed. The anti-erosive toothpaste Elmex Protection Erosion and toothbrush Elmex Erosion Soft promise to protect from dental erosions/abrasions and have been used in multiple related studies.^2,13,33,36^ However, to our knowledge, there are no studies comparing different combinations of specific anti-erosive and conventional toothpastes/-brushes in terms of interaction and compatibility.

Therefore, it was the aim of this study to investigate erosive/abrasive enamel wear of previously eroded enamel while using a combination of anti-erosive and standard toothbrushes/-pastes. The null hypothesis was that the erosive/abrasive potential of the different combinations does not differ.

## Material and Methods

### Specimen Preparation and Allocation

**Fig 1:**
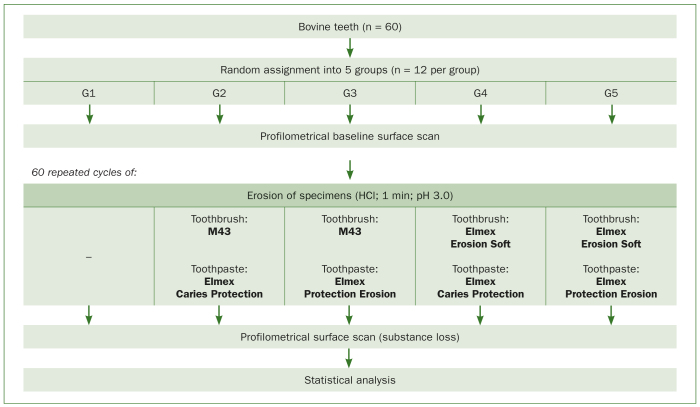
Experimental design.

The experimental design is illustrated in Figure 1. A total of 60 enamel specimens were gained from the crowns of extracted permanent bovine lower incisors stored in tap water (Zurich, no added fluoride) until use. Cylindrical enamel specimens (3 mm diameter) were prepared using a diamond trephine mill (BFW 40/E, PROXXON; Föhren, Germany) and centrally embedded in acryl resin (Paladur, Kulzer; Hanau, Germany) in order to enable sufficient fixation during profilometric surface scan. In an automatic grinding machine with 5 N pressure, 150 U/min and water cooling (Tegramin 30, Struers; Birmensdorf, Switzerland), specimens’ enamel surfaces were ground flat in three steps using carborundum discs (Water Proof Silicon Carbide Paper, Struers) with decreasing grain size (1200 grit, 5 s; 2000 grit, 40 s; 4000 grit, 120 s). Subsequently, the prepared specimens were randomly assigned into five groups (G1–G5) of 12 specimens each, numerically labelled, and stored in tap water.

### Erosive/Abrasive Treatment

**Fig 2:**
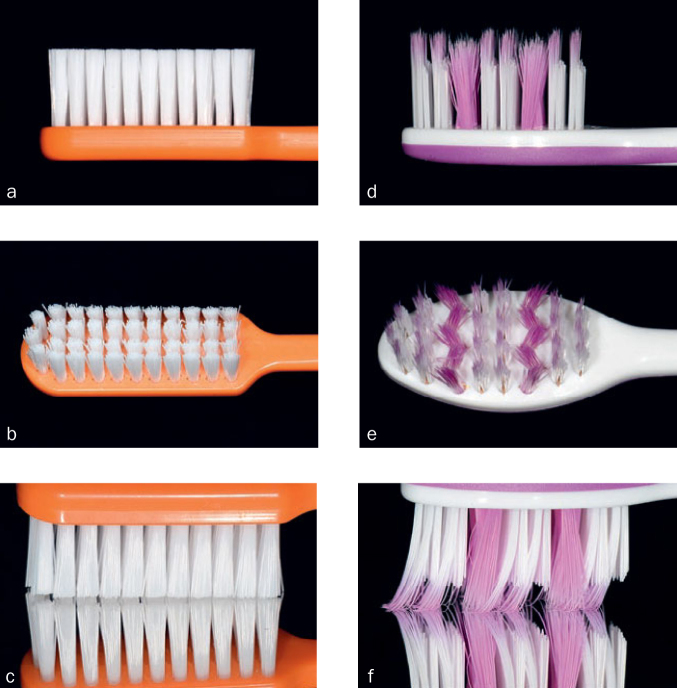
Images of the standard medium toothbrush Paro M43 (a–c) and the anti-erosive toothbrush Elmex Erosion Soft (d–f) from different angles. (c) and (f) show the deformation of the bristles under load (200 g).

Prior to erosive and abrasive treatment, the surface baseline profile of each specimen was recorded. In a total of 60 cycles, specimens then were eroded by applying 5 ml hydrochloric acid (HCl) (pH 3.0) with a pipette for 60 s, rinsed with tap water to stop the erosive attack and then each group subsequently treated with the different combinations of toothbrushes (Paro M43, Esro; Kilchberg, Switzerland; Elmex Erosion Soft, GABA; Therwil, Switzerland) and toothpastes (Elmex Caries Protection, GABA; Elmex Protection Erosion, GABA). The allocations of the different toothbrush/-paste combinations and control group are given in Figure 1. Further detailed information about the toothbrushes and -pastes used in this study are given in Table 1 and images of the brushes from different angles are illustrated in Figure 2. Specimens of all groups, except for the control group, were brushed with the associated toothpaste slurry (mix of toothpaste and artificial saliva at a ratio of 1:3)^20^ and associated toothbrush. While performing 15 brushing strokes per cycle, a constant brushing force of about 2.0 N was applied by fixing a 200 g weight on the head of the toothbrush. Images (c) and (f) in Figure 2 show the deformation of the bristles under the applied load. Following each brushing, specimens were rinsed with tap water before they were gently dried with compressed air for 10 s and re-exposed to HCl to start the next cycle.

**Table 1:** Information and composition of the toothbrushes and toothpastes used in the present study

Toothbrush	Information	Manufacturer
Paro M43	Bristles total: n = 1548 per tuft: n = 36 thickness: 0.2 mm length (plane): 11 mm	Esro; Kilchberg, Switzerland
Elmex Erosion Soft	Bristles total: n = 1600 per tuft: n = 50 thickness: 0.18 mm length (bilevel): 8.2–10.2 mm (white); 10.0–12.4 mm (white/purple); 8.8–12.0 mm (purple)	GABA; Therwil, Switzerland
Toothpaste	Composition	Manufacturer
Elmex Caries Protection	Aqua, Hydrated Silica, Sorbitol, Hydroxyethylcellulose, Olaflur (1400 ppm), Aroma, Saccharin, Limonene, CI 77891 (Status 04.01.2016)	GABA; Therwil, Switzerland
Elmex Protection Erosion	Aqua, Hydrated Silica, Glycerin, Sorbitol, Hydroxyethylcellulose, Aroma, Cocamidopropyl Betaine, Olaflur (700 ppm), Sodium Gluconate, Stannous Chloride (3500 ppm Sn^2+^), Alumina, Chitosan (0.5%), Sodium Saccharin, Sodium Fluoride (700 ppm), Potassium Hydroxide, Hydrochloric Acid, CI 77891 (Status 04.01.2016)	GABA; Therwil, Switzerland

### Profilometric Analysis

Before baseline profiles were recorded, two parallel reference lines (distance 3.4 mm) were notched in the embedding acryl resin of each specimen close to the enamel margin. Additionally, profilometer and specimens were equipped with a jig to ensure exact repositioning. From each specimen five baseline profiles with a distance of 100 μm between each profile were recorded using a stylus profilometer (Perthometer S2 Concept, Mahr; Göttingen, Germany) with a stylus force of <0.7 mN and a lower measuring limit of <130 nm profile difference.^39^ After 60 test cycles, surface profiles were recorded again and enamel wear caused by erosion (G1–5) and abrasion (G2–5) was calculated using a custom made software able to perform superimposition of the baseline profiles and follow-up profiles. Superimposition of the two profiles was achieved by overlaying the reference areas (area outside the two reference lines). The step height between the baseline profile and follow-up profile in the area of the treated surface was considered as enamel wear. In case the assessed wear per profile was below the measurement limit of the profilometer (0.105 μm),^1^ the value for this profile was set to 0.000 μm. While the enamel wear of each specimen was calculated by averaging the values of the five respective profiles, enamel wear of each of the five groups was gathered by averaging the values of the twelve specimens of the associated group.

### Statistical Analysis

As part of the descriptive statistics, means and standard deviation for enamel loss were computed. Additionally, data were analysed with two-way ANOVA including an interaction effect and residuals were checked for normality and variance homogeneity. T tests were applied to compare G2–5 to the control group G1. For post-hoc pairwise comparison, p values were corrected according to Tukey HSD (honest significant difference). The level of statistical significance was set at 5%. All statistical analyses and plots were done with the statistical software R version 3.2.2 (The R Foundation for Statistical Computing, Vienna, Austria; www.R-project.org).

## Results

**Fig 3:**
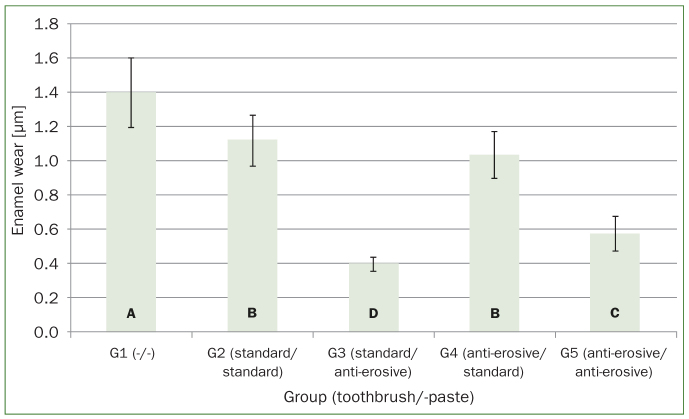
Enamel wear (µm) (mean ± SD) after 60 cycles of erosion (G1) or erosion and abrasion (G2–5) with a medium standard (Paro M43) or anti-erosive (Elmex Erosion Soft) toothbrush and a standard (Elmex Caries Protection) or anti-erosive (Elmex Protection Erosion) toothpaste. Values that are not statistically significantly different are marked with same capital letters.

The enamel wear of each group after 60 cycles of erosion or erosion and abrasion is illustrated in Figure 3. All five test groups showed more or less severe enamel wear. Except for G2 and G4 (p = 0.28), the pairwise t test with adjusted p value for multiple comparison revealed statistically significant differences between all groups (p < 0.01). The highest loss of enamel was observed in the control group G1 (1.4 ± 0.20 µm) where specimens were solely treated with HCl and not brushed. All four toothbrush/-paste combinations (G2–5) revealed significantly less enamel wear compared to the control group. G2 turned out to be the most abrasive toothbrush/-paste combination (1.12 ± 0.15 µm), G3 the least invasive (0.40 ± 0.04 µm). The kind of toothbrush and toothpaste both showed statistically significant influence on the resulting enamel wear (p < 0.001). Besides, the interaction between toothpaste and toothbrush also was statistically significant (p < 0.001) resulting in more or less severe enamel wear within the different combinations of these two parameters.

## Discussion

The results of the present study reveal significantly reduced enamel wear for all toothbrush/-paste combinations in comparison to the control group. Nevertheless, differences in the erosive/abrasive enamel wear between the respective combinations were found. Thus, the null hypothesis was rejected.

Specimens in this study were prepared from bovine teeth, which have been used in multiple studies investigating erosive and abrasive loss of dental hard tissues and can be regarded as a suitable replacement for human teeth.^4,6,26,29^ However, enamel loss in bovine teeth caused by erosion and abrasion might be slightly higher compared to human enamel.^6^ Nevertheless, bovine teeth provide several advantages as they can easily be obtained in great numbers with high homogeneity due to animal husbandry under similar conditions. Usually they are free of caries, other surface anomalies and multiple specimens can be gained from a single tooth^34,37^ thus allowing for homogenous specimens and a high compatibility of results.^40^

Specimens were stored in tap water and air dried just before profilometric analysis, which was shown to have no influence on the results.^1^ Profilometric surface analysis is proven to be a reliable and accurate method in order to quantify erosive/abrasive enamel wear.^10,32,38^ Without interference, the profilometer used in this study enables reproducibility of 0 ± 0.031 µm and a lower limit of detection of 0.105 µm.^1^ Duration and implementation of erosion (60 s) and abrasion (15 manual brushing strokes, 2 N brushing force) were based on recommendations by Wiegand and Attin^40^ aiming to simulate preferably realistic clinical conditions. However, due to the focus on abrasiveness of anti-erosive toothbrush/-paste combinations after erosion, remineralisation periods between erosion and abrasion were not taken into account in this study. On the one hand, those periods might enhance the abrasive wear resistance due to stabilisation of the previously eroded enamel; on the other hand, previous studies describe only a minor effect^14^ and increased abrasion of previously eroded enamel even after a remineralisation period of 1 h.^3^ An additional group in which specimens get eroded and brushed without any toothpaste was considered to be unsuitable to the study design (investigation of the interplay between different combinations of toothbrush and toothpaste) and to have little relevance in daily routine as it can be assumed, that most people use some sort of toothpaste while brushing their teeth. Another limiting factor might be that most people do not brush their teeth after each consumption of acidic food or beverages as it was performed in this study, but only one to two times a day. The resulting disproportion of erosion and abrasion was not respected in this study. Furthermore, physiological factors that might have an alleviating effect on erosion and/or abrasion, such as consistency and flow rate of saliva or the salivary pellicle were also disregarded in this in vitro study.

In this study, the kind of toothbrush as well as the kind of toothpaste had a statistically significant influence on the resulting wear of enamel. The anti-erosive toothpaste in combination with both kinds of toothbrushes (G3 and G5) showed significantly less enamel wear compared to the standard toothpaste (G2 and G4). The two major relevant parameters of a toothpaste concerning abrasion of eroded enamel are reported to be abrasiveness and fluoride content.^41^ The abrasive potential of a toothpaste correlates with its relative dentine abrasion (RDA),^17^ in particular being influenced by sort, size and shape of the abrasive particles.^23^ A recent study reported an RDA value of 57.4 ± 5 for Elmex Caries Protection and 18.8 ± 3 for Elmex Protection Erosion.^8^ It might be assumed that the differences in RDA between the two investigated toothpastes had a major influence on the significantly reduced enamel wear in case the anti-erosive toothpaste was used. Besides, in vitro and in situ studies show less wear of enamel and dentine in case a fluoride containing toothpaste is used,^4,40^ which is in conformity with the results of this study where the four fluoride containing test groups (Elmex Caries Protection: AmF 1400 ppm; Elmex Protection Erosion: AmF 700 ppm + NaF 700 ppm) revealed significantly less enamel wear compared to the control group. Not least, stannous chloride- and chitosan-containing toothpastes, such as Elmex Protection Erosion, have been reported in both in vitro and in situ studies to provide additional protection of enamel against erosive attacks.^13,15,33^ It might be suspicious that the control group showed the overall highest wear of enamel although no brushing was performed. An additional control group in which specimens were brushed with a non-fluoridated toothpaste after erosion most likely would have resulted in even higher enamel wear as it was shown in a study by Attin et al.^5^ The 60 repeated cycles of brushing with either a fluoridated or a fluoride-, stannous chloride- and chitosan-containing toothpaste in the test groups most likely enhanced the described protective effect of the respective toothpastes on eroded enamel.^13,14,27^

The abrasive potential of a toothbrush is influenced by brushing force, along with the material, shape and rigidity of the bristles. The significantly better performance – respectively, the lower loss of enamel in G3 (standard medium toothbrush Paro M43 and anti-erosive toothpaste Elmex Protection Erosion) compared to G5 (anti-erosive toothbrush Elmex Erosion Soft and anti-erosive toothpaste Elmex Protection Erosion) – might particularly be attributed to the different rigidities of the respective toothbrush bristles. Prematurely, one might assume that a softer bristled anti-erosive toothbrush might cause less wear of dental hard tissue compared to a harder bristled one due to its thinner taper and more flexible bristles. However, and especially because of these properties, they seem to be able to carry more toothpaste, spread it on a larger surface and create longer huddling surface contact. Thus, the longer and more extensive interaction between toothpaste and enamel surface might lead to higher abrasive enamel wear compared to the standard toothbrush. This assumption is in conformance with the findings of a recent study by Bizhang et al, where abrasive loss in dentine was higher with soft-bristled compared to hard-bristled toothbrushes.^12^ Nevertheless, enamel wear in G5 was still significantly reduced compared to the two groups using the standard toothpaste (G2 and G4). Regarding those two groups, no statistically significant difference between standard and anti-erosive toothbrush was observed in case the standard toothpaste was applied, which emphasises the potentially greater influence of toothpastes on enamel wear.

## Conclusion

Within the limitations of the present study it can be concluded that all tested combinations of toothbrushes/-pastes are able to significantly reduce erosive/abrasive enamel wear. The combination of a standard toothbrush and anti-erosive toothpaste appears to be the least invasive combination. Nevertheless, none of the tested combinations is able to completely avoid erosive/abrasive enamel wear implying the need of comprehensive instructions of compromised patients and additional preventive interventions.
